# Restricted maternal nutrition and supplementation of propylene glycol, monensin sodium and rumen‐protected choline chloride during late pregnancy does not affect muscle fibre characteristics of offspring

**DOI:** 10.1002/vms3.1239

**Published:** 2023-08-09

**Authors:** Leila Ahmadzadeh‐Gavahan, Ali Hosseinkhani, Gholamreza Hamidian, Seyedhosein Jarolmasjed, Reza Yousefi‐Tabrizi

**Affiliations:** ^1^ Department of Animal Science, Faculty of Agriculture University of Tabriz Tabriz Iran; ^2^ Department of Basic Sciences, Faculty of Veterinary Medicine University of Tabriz Tabriz Iran

**Keywords:** feed restriction, monensin sodium, muscle fibre characteristics, propylene glycol, rumen‐protected choline chloride

## Abstract

**Background:**

Grazing in arid and semi‐arid regions faces pregnant ewes with feed restrictions and hence affects the offspring muscle fibre characteristics. Using feed additives that enhance nutrient availability during foetal muscle development is expected to alter offspring skeletal muscle characteristics.

**Objectives:**

This study evaluated the effect of maternal restricted nutrition and supplementation of propylene glycol, monensin sodium and rumen‐protected choline chloride on lamb's muscle fibre characteristics.

**Methods:**

Forty‐eight Ghezel ewes were randomly allocated to one of six diets (*N* = 8) during the last 6 weeks of gestation: ad libitum feed intake (AL); restricted feeding (RF); restricted feeding containing propylene glycol (PG); restricted feeding containing propylene glycol and monensin sodium (MS); restricted feeding containing propylene glycol and rumen‐protected choline chloride (RPC); restricted feeding containing propylene glycol, monensin sodium and rumen‐protected choline chloride (PMC). The muscle samples were obtained from the semitendinosus muscle of 2‐week‐old male lambs (*n* = 5/treatment) via biopsy and were stained and classified as fibre types I, IIA and IIB.

**Results:**

Pre‐parturient maternal feed restriction and administration of propylene glycol, monensin sodium and rumen‐protected choline chloride had no significant effect on fibre‐type composition, fibre density of muscle, muscle cross‐sectional area and volume density of fibres (*p* > 0.05).

**Conclusions:**

Either maternal dietary restriction or supplementation of nutrient flux–involved additives during late pregnancy did not alter muscle fibre development and had no short‐term effects on muscle properties of the resulting offspring as myogenesis occurs in early and mid‐gestation, not late gestation. Therefore, maternal nutrition may not be a problematic issue in sheep production in arid and semi‐arid areas.

## Graphical abstract

1. Grazing in arid and semi‐arid regions causes ewes to face some degree of feed restriction during late pregnancy when the offspring muscle fibres are formed.

2. We hypothesized that the supplementation of feed‐restricted diet with additives involved in increased nutrient delivery to the dam during foetal muscle development will overcome the compromised effects of feed restriction.

3. Maternal feed restriction and combined supplementation of propylene glycol, monensin sodium and rumen‐protected choline chloride did not affect offspring muscle fibre characteristics, including fibre‐type composition, fibre density of muscle, muscle cross‐sectional area and volume density of fibres.

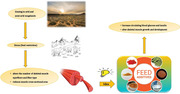

## 1 INTRODUCTION

Investigating muscle fibre characteristics is important for meat scientists, breeders and the meat industry in order to get a better knowledge about the role of muscle fibres in muscle growth and final meat quality characteristics (Wegner et al., 2000). For example, the effects of histochemical characteristics on meat tenderness, water‐holding capacity, juiciness or fat content have been previously demonstrated (Lyczynski et al., 2009; Rehfeldt et al., 2000). Moreover, it is widely accepted that muscle fibre composition is an important source of variation in meat quality (Javaid et al., 2012).

Muscle fibres are the basic structural units of skeletal muscle. They account for over 75% of muscle volume. Therefore, the morphology of muscle fibres (the number and size of muscle fibres) is the major determinant of skeletal muscle mass (Lee et al., 2010). Various classification methods are used to classify muscle fibres into different types. A commonly used method is that of Brooke and Kaiser (1970), which classifies muscle fibres into three types (I, IIA and IIB) based on their pH sensitivity to myosin adenosine triphosphatase (mATPase) activity. The metabolic, structural and contractile properties of individual fibre types (Choi and Kim, 2009) are different, and these differences can affect meat quality and carcass traits in different animal species (Lefaucheur, 2010; Kim, Jeong, et al., 2013; Bogucka and Kapelanski, 2016). On the other hand, the qualitative and quantitative parameters of carcass traits are usually related to muscle fibre characteristics, such as the total number of fibres, fibre density, fibre cross‐sectional area and fibre type composition in muscle (Joo et al., 2013; Kim et al., 2014). For example, higher proportions and larger areas of glycolytic type IIB fibres in the muscle are associated with lighter meat and lower water holding capacity (Ryu et al., 2006; Lefaucheur, 2010; Kim, Kim, et al., 2013). In the study of Nam et al. (2009), the cross‐sectional area and the area and the number of percentages of type I muscle fibres were positively correlated with the colour acceptability of fresh meat and the taste of cooked meat.

There are many factors that affect the characteristics of muscle fibres, including breed, gender and age (Rehfeldt et al., 2000; Jeong et al., 2012; Joo et al., 2013). One of the most important factors is nutrition (Bee et al., 2007; Jeong et al., 2012). In arid and semi‐arid regions such as Iran, the sheep production system is highly dependent on the natural vegetation of ranges and farmlands. Therefore, animals can be naturally submitted to periods of feed restriction due to seasonal fluctuations, causing a periodical restriction in food quality and quantity. As the formation of muscle fibres begins around day 20 and is completed between days 80 and 125 of pregnancy (Ashmore et al., 1972; Maier et al., 1992) and there is no further increase in the number of these fibres after birth (Du et al., 2010) and considering the profound effects of prenatal muscle fibre numbers on muscle growth and development in post‐natal life, any feed restriction occurring during pregnancy may alter muscle development (Dauncey and Harrison, 1996), alter the number of skeletal muscle myofibres and fibre type and reduce muscle cross‐sectional area (Reed et al., 2014; Du et al., 2015) in the offspring. Changes in muscle fibre composition can, in turn, relatively predict the walking ability of grazing animals that have to run long distances to get feed to meet their nutritional requirements in arid and semi‐arid regions. It is well known that muscle fibre composition with a greater proportion of type I fibres is associated with success in slower, longer distance events (Tesch and Karlsson, 1985). Therefore, the determination of muscle fibre composition will allow us to decide about their long‐distance walking ability.

There are some feed additives that can alleviate the implications of maternal feed restriction during late pregnancy. Some of the most well‐known and extensively studied additives are propylene glycol, monensin sodium and rumen‐protected choline chloride. These additives can manipulate maternal endocrine status or energy metabolism pathways by raising circulating blood glucose and insulin secretion (Ahmadzadeh‐Gavahan et al., 2021). As skeletal muscle is particularly vulnerable to nutrient availability during foetal development, the supplementation of these additives is expected to alter skeletal muscle protein synthesis and programming of offspring muscle (Davis and Fiorotto, 2009) via increased maternal nutrient supply for the normal development of the fetus.

To date, there is no evidence of any immediate outcomes of late gestational poor maternal nutrition or supplementation of propylene glycol, monensin sodium and rumen‐protected choline chloride on offspring muscle fibre characteristics in Ghezel sheep. Therefore, the main objective of this study was to investigate the immediate impacts of maternal feed restriction and supplementation of the abovementioned additives during the last 6 weeks of prepartum on fibre characteristics in the semitendinosus muscle of resulting Ghezel lambs in order to predict their meat quality and quantity and also their walking ability.

## 2 MATERIALS AND METHODS

### 2.1 Experimental design, animals and treatments

Ninety‐six, 2–3‐year old Ghezel ewes were chosen to be assessed in the present study. Briefly, progesterone sponges (Ponjavet, Hipra) containing 60 mg of medroxyprogesterone acetate were inserted intravaginally, removed after 14 days and ewes were then received a single intramuscular injection of 500 IU of PSMG. Two days after sponges were expelled, all ewes were monitored for oestrus signs twice daily and then introduced to Ghezel rams for natural breeding. The study only included ewes who did not show signs of second oestrus 17–20 days after mating and became pregnant after the first oestrus. Therefore, 48 multiparous Ghezel ewes (BW 65.53 ± 6.90 kg and BCS 3.17 ± 0.56) were investigated in a completely randomized design. All ewes grazed on local pastures under extensive conditions after mating (day 0 of pregnancy). Transabdominal ultrasonic scanning (SIUI CTS‐900V) was used to confirm pregnancy roughly 40 days after mating. By day 90 of pregnancy, singleton and twin fetuses carrying ewes (in an equal ratio; four twin and four singleton; Table 1) were equally (*N* = 8) assigned to the straw‐bedded pens and after 2 weeks’ adaptation to the experimental situation, diets were fed as follows: ad libitum feed intake (AL); restricted feeding (RF, 60% feed restriction); restricted feeding containing propylene glycol (67 g/day) (PG); restricted feeding containing propylene glycol and monensin sodium (30 mg/day) (MS); restricted feeding containing propylene glycol and rumen‐protected choline chloride (6 g/day) (RPC); restricted feeding containing propylene glycol, monensin sodium and rumen‐protected choline chloride (PMC). Feed restriction was achieved by allocating the restricted dams 60% of AL to simulate a seasonal feed deficiency. In order to determine the impact of feed restriction on ketosis induction, all ewes were checked for ketosis signs and confirmed to be healthy (Ahmadzadeh‐Gavahan et al., 2021). Experimental additives, including monensin sodium (10%), propylene glycol (45%) and rumen‐protected choline chloride (25%), were provided by Behrood Atrak Co., Difagri Co. and SILA Co., respectively. The applied dosages of propylene glycol, monensin sodium and rumen‐protected choline chloride were based on previously published literature in dairy ewes or goats (Ahmadzadeh‐Gavahan et al., 2018; Hajikhajehlou et al., 2010; Pinotti et al., 2008) and manufacturer's recommendations. The diets were formulated with a roughage‐to‐concentrate ratio of 65:35 to meet the nutritional requirements of pregnant ewe (National Research Council (NRC), 2007) based on their litter size and stage of pregnancy. All of the animals were housed and fed in a group while the feed allocated to the twin or singleton bearing ewes was separated from each other within a treatment pen. The maximum space requirements of a trough for each ewe (33–50 cm) were assigned to ensure that all animals can eat equally without any competition in the feeding process. Table 2 shows the daily nutrient intake of the ewes during pre‐ and post‐partum periods. After feed restriction was withdrawn at parturition, all ewes were offered post‐partum diet as AL. During the adaptation period, animals’ feed intake was determined using the difference between the feed offered to the ewes on the previous day and the feed rejected, with about 10% of the total remaining as refusals (Laporte‐Broux et al., 2011). The dry matter intake of the ewes was determined by daily monitoring of the feed offered and refused. As previously published, the actual mean dry matter intake of ad libitum‐fed ewes was 2.14 kg DM/day, whereas that of restricted ewes was 1.35 kg DM/day (Ahmadzadeh‐Gavahan and Hosseinkhani, 2022). Daily feed samples were collected before feeding at 08:30 h and, after weighing, were homogenized, sub‐sampled and stored at −20°C until analysis for chemical composition according to the Association of Official Analytical Chemists (AOAC) (1995) procedures. Offspring born to the abovementioned dietary groups are referred to as AL, RF, PG, MS, RPC and PMC hereafter.

**TABLE 1:** Birth type and sex of offspring in different dietary groups.

Trait	AL	RF	PG	MS	RPC	PMC
Birth type						
Single	4	4	4	4	4	4
Twin	4	4	4	4	4	4
Sex						
Male	5	8	5	6	9	8
Female	7	4	7	6	3	4

Abbreviations: AL, ad libitum feed intake; MS, restricted feeding containing propylene glycol and monensin sodium; PG, restricted feeding containing propylene glycol; PMC, restricted feeding containing propylene glycol, monensin sodium and rumen‐protected choline chloride; RF, restricted feeding; RPC, restricted feeding containing propylene glycol and rumen‐protected choline chloride.

**TABLE 2:** Formulation and chemical composition of the basal diet.

Item	Diet composition	
	Prepartum	Post‐partum
Ingredient (g/kg DM)		
Corn silage	330	200
Alfalfa hay	190	290
Wheat straw	140	110
Ground barley grain	160	132
Wheat bran	122	150
Molasses	50	60
Soybean meal	0	50
Salt	3	3
Mineral–vitamin premix^a^	5	5
Chemical composition		
Dry matter (% of fresh weight)	73	78.90
Metabolizable energy^b^ (MJ/kg DM)	9.37	9.66
Crude protein (g/kg of DM)	100.65	127.02
Calcium (g/kg of DM)	4.37	5.48
Phosphorus (g/kg of DM)	3.46	4.06
Dry matter intake (kg/day)	2.4	1.9

^a^ Ingredients per kg included 500,000 IU vitamin A, 100,000 IU vitamin D3, 100 mg vitamin E, 196,000 mg calcium, 96,000 mg phosphorous, 19,000 mg magnesium, 46,000 mg sodium, 2000 mg manganese, 3000 mg iron, 300 mg copper, 3000 mg zinc, 100 mg cobalt, 100 mg iodine, 1 mg selenium, 400 mg butylated hydroxytoluene oxide.

^b^ Estimated using values got from the National Research Council (2007).

### 2.2 Sample collection

Five male lambs from each treatment group (*n* = 5; BW 5.67 ± 1.16) were selected for sample collection on day 14 after birth and the remaining lambs stayed with their dams and returned to the flock. Because males are often slaughtered for market and females are maintained for breeding, the male lamb was chosen for the study. At biopsy, lambs were anaesthetized with an intravenous injection of xylazine (0.1 mg/kg BW), and samples of left semitendinosus muscle were collected from the midpoint of the muscle using a surgical punch at 0.5 × 0.5 × 0.3 cm^3^ and fixed in 10% neutral buffered formalin for further analysis.

### 2.3 Histochemical analysis

**FIGURE 1:**
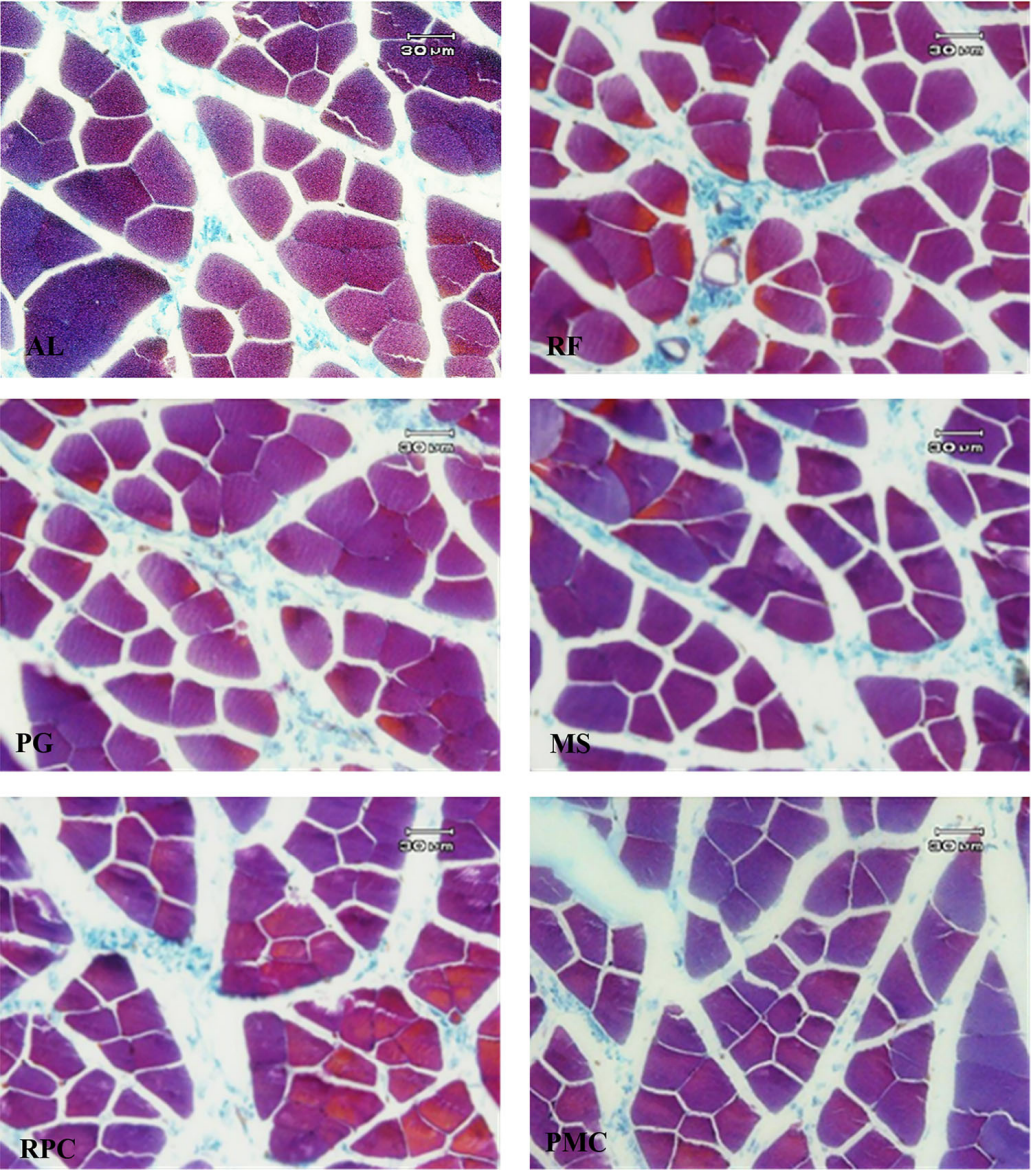
Photomicrographs of semitendinosus muscle of 2‐week‐old lambs (Mallory's trichrome staining, ×400). AL, ad libitum feed intake; RF, restricted feeding; PG, restricted feeding containing propylene glycol; MS, restricted feeding containing propylene glycol and monensin sodium; RPC, restricted feeding containing propylene glycol and rumen‐protected choline chloride; PMC, restricted feeding containing propylene glycol, monensin sodium and rumen‐protected choline chloride. Bar = 30 μm.

The routine and standard method of paraffin embedding was used for histochemical analysis. For this purpose, about 7 μm of excised serial sections after rehydrating by a graded series of incubations in xylene and ethanol solutions were embedded in paraffin and cut into sections using a rotary microtome. Tissue sections were stained with Mallory's trichrome for analysis by standard light microscopy (Figure 1). All morphometric studies were performed using a version 9 stereo‐investigator system (MBF Bioscience, Micro Bright Field, Inc.) on at least 10 different microscopic fields of each sample. The mean cross‐sectional area of 20 fibres (μm^2^) per section and the number of fibres per mm^2^ (fibre density) were quantified for each section. The volume density (*V_v_*) of muscle fibres in tissue samples was estimated by a point‐counting method and using the M_42_ test grid in a stereo‐investigator system (Eisenberg et al., 1974; Novaes et al., 2017).

### 2.4 Muscle fibre typing

The histochemical evaluation was conducted to identify muscle myofibre types. For this purpose, serial cross sections (10 μm thickness) of each sample were obtained at −25°C using a Leica CM 1850 cryostat microtome, mounted on coverslips and stained for myosin ATPase (mATPase) activity. This staining method was based on that of Brooke and Kaiser (1970) in which sections were reacted for actomyosin ATPase stability after successive alkaline (pH 10.4) and acid (pH 4.35 and 4.6) preincubation. Fibres were classified according to their contractile properties as type I (the darkest, slow‐twitch oxidative fibres), type IIA (medium, fast‐twitch oxido‐glycolytic fibres) and type IIB (light, fast‐twitch glycolytic fibres) based on the nomenclature of Brooke and Kaiser (1970). The serial sections were viewed using a stereo‐investigator system, and at least five areas were selected randomly for each sample and the numbers of fibres of each type within a known area were counted (Tamaki et al., 2014).

### 2.5 Statistical analysis

The data were analysed as a completely randomized design using UNIVARIATE and GLM procedures of SAS v.9.3.1 (SAS Institute Inc., 2011). The muscle fibre characteristics and histological data were evaluated by three‐way ANOVA, with maternal feeding regimen, parity and litter size as the fixed effects and the body weight of lamb and dam as covariates. Residuals from all analyses were examined to confirm their normality. Where applicable, multiple comparisons of means were performed using the LSMEANS statement of the GLM procedure and the Tukey–Kramer multiple comparison method. Data are presented as least square means and standard error of means and significance level *p* < 0.05 was used to evaluate the differences between dietary groups.

## 3 RESULTS AND DISCUSSION

**FIGURE 2:**
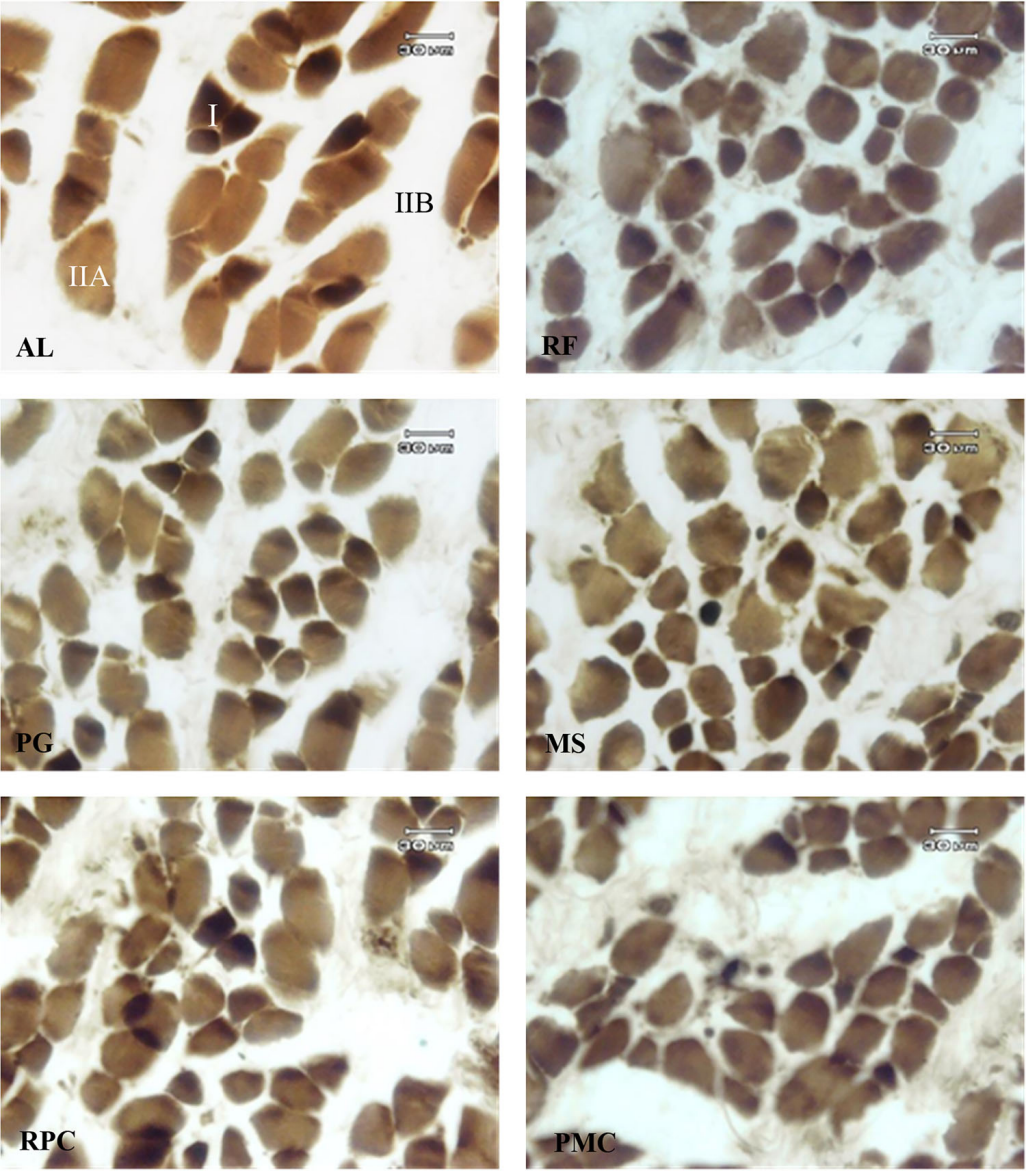
Fibre typing by staining for ATPase activity after alkaline preincubation on the cross section of semitendinosus muscle of 2‐week‐old lambs (×400). AL, ad libitum feed intake; RF, restricted feeding; PG, restricted feeding containing propylene glycol; MS, restricted feeding containing propylene glycol and monensin sodium; RPC, restricted feeding containing propylene glycol and rumen‐protected choline chloride; PMC, restricted feeding containing propylene glycol, monensin sodium and rumen‐protected choline chloride. ‘I’ is the slow‐twitch oxidative fibre (dark brown); ‘IIA’ is the fast‐twitch oxido‐glycolytic fibres (light brown); ‘IIB’ is the fast‐twitch glycolytic fibres (white) (Brooke and Kaiser, 1970). Bar = 30 μm.

Muscle fibres of type I, IIA and IIB, which were identified in semitendinosus muscle sections using the alkaline ATPase technique, are presented in Figure 2. The primary myofibres are large, with the nucleus located at the centre of the fasciculus appearing as a dark spot on fluorescent microscopy. Secondary myofibres, which are smaller and had a peripherally located nucleus, surround each primary myofibre.

As shown in Table 3, the animals in all groups presented a higher proportion of type I fibres, followed by type IIB fibres, with a lower percentage of type IIA fibres. Interestingly, no effect of prepartum maternal dietary treatments was noted on the fibre type of semitendinosus muscle samples obtained from six groups of lambs (*p* > 0.05). This result is in accordance with that reported by Fahey et al. (2005) who showed no alteration in muscle fibre type of 14‐day‐old lambs born from previously undernourished ewes (dietary restriction during days 55–95 and 85–115 of pregnancy). Similarly, Krausgrill et al. (1999) also found that maternal nutrient restriction did not influence the percentage of muscle fibre types at any of the ages studied (70‐ and 140‐day fetus). In contrast, Zhu et al. (2004) found fewer muscle secondary myofibres in the fetus (78 days) that were undernourished in utero during days 28–78 of gestation compared with controls, which may be related to the reduced nuclear proliferation (Madgwick, 1991).

**TABLE 3:** Effect of feed restriction and supplementation of propylene glycol, monensin sodium and rumen‐protected choline chloride on fibre characteristics of semitendinosus muscle of 2‐week‐old male lambs.

	Feeding groups						
Trait	AL	RF	PG	MS	RPC	PMC	*p* Value
Mean cross‐sectional area (μm^2^)	1013.00 ± 23.47	1040.25 ± 34.08	1018.00 ± 27.88	1060.75 ± 32.50	978.67 ± 24.14	1025.00 ± 31.64	0.52
Volume density of fibres (mm^2^)	67.17 ± 1.33	65.50 ± 2.25	65.83 ± 1.47	64.25 ± 1.93	72.33 ± 4.85	65.50 ± 1.45	0.33
Fibre density (mm^2^)	646.83 ± 7.49	635.75 ± 7.85	641.83 ± 6.73	634.00 ± 7.63	655.17 ± 8.01	639.67 ± 6.85	0.41
Fibre type composition (%)							
Type I	44.83 ± 1.66	46.25 ± 2.36	46.17 ± 1.96	49.25 ± 1.49	48.83 ± 2.02	46.50 ± 1.23	0.52
Type IIA	14.33 ± 0.80	13.75 ± 1.03	14.33 ± 1.05	13.00 ± 0.41	13.17 ± 0.83	14.67 ± 0.76	0.70
Type IIB	40.83 ± 1.45	40.00 ± 1.68	39.50 ± 1.06	37.75 ± 1.11	38.00 ± 1.41	38.83 ± 1.22	0.58

Abbreviations: AL, ad libitum feed intake; RF, restricted feeding; PG, restricted feeding containing propylene glycol; MS, restricted feeding containing propylene glycol and monensin sodium; RPC, restricted feeding containing propylene glycol and rumen‐protected choline chloride; PMC, restricted feeding containing propylene glycol, monensin sodium and rumen‐protected choline chloride.

On the other hand, other authors (Zhu et al., 2006; Daniel et al., 2007) observed a higher proportion of IIB myofibres in lambs born from restricted fed ewes in comparison to those born from ad libitum fed group. A higher proportion of IIB fibres could be associated with greater lean meat content, which is closely related to poor meat quality. As there were no significant differences in muscle type IIB fibres among AL, RF, PG, MS, RPC and PMC lambs, meat quality traits would not be different in these groups, and they would have similar lean meat content. Moreover, due to similar type I fibres, lambs in all groups will have similar walking abilities during grazing, as the correlation between long‐distance activity and type I fibres has been previously demonstrated (Tesch and Karlsson, 1985).

Similar fibre‐type composition among all groups of lambs may also suggest that although muscle fibre development occurs prenatally, reduced maternal feeding during gestation and supplementation of propylene glycol, monensin sodium and rumen‐protected choline does not affect fibre‐type composition which may be due to similar optimal diet post‐natally (Khan et al., 2013) enabling lambs to somehow compensate for the effects of maternal dietary restriction on fibre composition, resulting in no detrimental effects on muscle fibre characteristics. However, alterations in fibre‐type composition due to poor maternal diet are conflicting. The contrasting results are likely due to differences in species, the severity of restriction, the type of poor maternal diet, the timing and/or duration of exposure to poor maternal nutrition and age of offspring (Bee, 2004; Zhu et al., 2004; Woo et al., 2011; Yan et al., 2013; Tygesen et al., 2007). For instance, the cross‐sectional area of the semitendinosus muscle from offspring subjected to underfeeding is altered in comparison with controls, depending on the stage of post‐natal growth (Reed et al., 2014).

No difference was observed for the mean cross‐sectional area of fibres among AL, RF, PG, MS, RPC and PMC lambs (*p* > 0.05). Similarly, He et al. (2020) showed that muscle fibre cross‐sectional area was not altered in the pigs at birth with maternal MET (methyl‐donor micronutrients, such as folic acid, methionine, choline and betaine) dietary supplementation. In contrast to our results, Reed et al. (2014) reported that poor maternal nutrition reduced the cross‐sectional area of muscles as early as within 24 h of birth and at 3 months of age compared with control lambs, supporting inhibited muscle growth, altered expression of genes involved in myogenesis and decreased the number of skeletal muscle precursor cells (Woo et al., 2011) as a result of poor maternal nutrition during gestation. Our observation confirms that a similar cross‐sectional area between six dietary groups will result in a similar loin area in these groups as Lee et al. (2016) reported a very high correlation coefficient between the loin area and the cross‐sectional area of all fibres (*r* = 0.92). The results of the analysis also showed that, by the end of gestation, maternal dietary treatments did not affect the volume density of muscle fibres from the semitendinosus muscle (*p* > 0.05). The myofibre volume density of lamb hind–limb muscles is probably not a trait that can be changed by the supplementation of dams during gestation, regardless of the period, as has been reported by Mexia et al. (2006) for the semitendinosus muscle of lambs.

When compared by the ANOVA test, the prepartum maternal feed restriction and supplementation of propylene glycol, monensin sodium and rumen‐protected choline chloride did not cause a significant difference in the fibre density of the lambs within nutritional treatments (Table 3), which is in line with the results of He et al. (2020) during maternal methyl‐donor micronutrient supplementation. It has been shown that lower birthweights are associated with a reduced total fibre number (Powell and Aberle, 1981). As we have previously published that lambs born from ad libitum fed ewes and those born from restricted fed ewes had a similar birthweight (Ahmadzadeh‐Gavahan et al., 2020), a similar muscle fibre number between the AL and RF lambs was expected. On the other hand, dam devises ways to protect her fetus from imposed maternal feed restriction, which results in maternal tissue loss, as previously reported (Ahmadzadeh‐Gavahan et al., 2021) to preserve normal fetus growth (Du et al., 2004). Stem‐cell activity and tissue development are controlled by energy metabolism and nutrient flux during pregnancy (Shyh‐Chang et al., 2013; Hu et al., 2016). Therefore, we speculated that supplementing the restricted diet with propylene glycol, monensin sodium and rumen‐protected choline chloride during pregnancy may enhance fibre density since the positive impact of these additives in providing nutrients for the dam that is needed for skeletal muscle differentiation and maturity in offspring has been previously demonstrated (Ahmadzadeh‐Gavahan et al., 2021), whereas we did not get this expected result. This may be due to the fact that skeletal muscle has lower priority during foetal programming, and nutrients received from the mother to the fetus are first partitioned to vital organs, such as the brain, liver and heart, before muscle (Yajnik, 2004). Therefore, it is reasonable that the supplementation of these additives could not alter the fibre characteristics of offspring. Nonetheless, we failed to measure the other organs’ development in offspring.

In conclusion, maternal feed restriction by 60% during the final trimester of pregnancy, when most muscle fibres are formed, did not compromise the muscle fibre properties of the resulting 2‐week‐old lambs. Thus, maternal nutrition during late pregnancy may not be a critical factor in determining muscle characteristics of offspring in arid and semi‐arid regions. However, further study is needed to better determine the impact of poor maternal nutrition and supplementation of these additives during late pregnancy on meat quality properties.

## AUTHOR CONTRIBUTIONS

*Investigation; conceptualization and design the study; methodology; carrying out trial work; writing original draft*: Leila Ahmadzadeh‐Gavahan. *Project administration; supervision; validation; review and editing*: Ali Hosseinkhani. *Methodology; formal and statistical analysis; review and editing*: Gholamreza Hamidian. *Methodology*: Seyedhosein Jarolmasjed *Carrying out trial work; data curation*: Reza Yousefi‐Tabrizi. All the authors have read and approved the final version of this manuscript.

## CONFLICT OF INTEREST STATEMENT

No potential conflict of interest was reported by the authors.

## FUNDING INFORMATION

The authors reported that there is no funding associated with the work featured in this article.

### ETHICS APPROVAL STATEMENT

All animal procedures were reviewed and conducted under the guidelines outlined by the Iranian Council of Animal Care reviewed in Secretariat of the Biomedical Ethics Committee of University of Tabriz (NO: 2048).

### ETHICS STATEMENT

The authors confirm that the ethical policies of the journal, as noted on the journal's author guidelines page, have been adhered to and the appropriate ethical review committee approval has been received. The US National Research Council's guidelines for the Care and Use of Laboratory Animals were followed.

### PEER REVIEW

The peer review history for this article is available at https://www.webofscience.com/api/gateway/wos/peer‐review/10.1002/vms3.1239.

## Data Availability

No.
